# A6 MECHANISMS UNDERLYING GUT DYSFUNCTION FOLLOWING C. *DIFFICILE* INFECTION AND IMPLICATIONS FOR TREATMENT

**DOI:** 10.1093/jcag/gwab049.005

**Published:** 2022-02-21

**Authors:** Z Saqib, X BAI, G De Palma, A Hynes, M Surette, P Bercik, S M Collins

**Affiliations:** 1 Medicine, McMaster University, Mississauga, ON, Canada; 2 Medicine, McMaster University, Hamilton, ON, Canada; 3 McMaster University, Hamilton, ON, Canada

## Abstract

**Background:**

Recent evidence suggests an increasing prevalence of gut dysfunction following C. *difficile* infection (CDI). The accompanying prolonged antibiotic (AB) exposure likely contributes to chronic gut dysfunction and our ability to induce gut dysfunction in germ free (GF) mice colonized with microbiota from a patient with severe slow transit post CDI (PCDI) supports this notion (10.1093/jcag/gwz047.117). Furthermore, we were able to restore gut function following fecal microbial transfer from healthy murine donors. Our studies have implicated a role for macrophages in the destruction of the Interstitial Cell of Cajal (ICC) network underlying slow colonic transit in the humanized mouse model. These findings prompted us to evaluate microbiota-directed therapy in normalising gut function in this model.

**Aims:**

**1**)To investigate whether dietary psyllium rescues the development of slow colonic transit (SCT) through modulating host function via microbiota mediated immune mechanisms; and **2**)To evaluate the mechanisms underlying the beneficial effects of psyllium

**Methods:**

GF mice were colonized with either microbiota from the PCDI patient or healthy control (HC) for 3 weeks following which PCDI mice were treated with either a control diet or a 15% psyllium diet (PSY). Colonic motility was assessed before and after the diet intervention using the bead expulsion test. Stool samples were collected for microbial profiling, and short and branched-chain fatty acids (SCFA/BCFA) analysis. Colonic muscle layers encompassing myenteric plexus (MP) were collected for gene expression analysis and to evaluate activated macrophages and ICC degeneration using immunohistochemistry.

**Results:**

Microbiota from a PCDI patient induced a SCT phenotype in GF mice (n=13) as compared to mice colonized with HC microbiota (p=0.0002). Psyllium rescued this SCT phenotype in mice (PCDI(n=7) vs.PSY(n=6):p=0.0014). The psyllium-induced rescue was accompanied by normalization of the ICC network and morphological alterations in infiltrating macrophages. This was supported by changes in immune-related gene expression in the MP including CD11b, NOS, Myd88, Mapk1 and NF-κB. Additionally, bacterial composition was different between PCDI and PSY group (p=0.003). SCFAs like acetic and propionic acid were increased, while BCFA like isobutyric and isovaleric acid were decreased following PSY treatment. These alterations in SCFA/BCFA were supported by fluctuations in specific bacteria like Butyricimonas, Phascolarctobacterium and Allistipes.

**Conclusions:**

Our results provide evidence that chronic gut dysfunction following CDI and AB exposure is microbiota-driven. Furthermore, microbiota-directed therapy using psyllium could serve as a novel therapeutic strategy to normalize gut function via microbiota-mediated restoration of immune homeostasis in these patients.

**Funding Agencies:**

W. Garfield Weston Foundation

